# Gut–brain axis dysfunction in Parkinson’s disease: a meta-analysis of microbiome dysbiosis and intestinal barrier markers

**DOI:** 10.3389/fmed.2026.1909087

**Published:** 2026-09-23

**Authors:** Pulkit Mehrotra, K. Vengadakrishnan, Puneet Mehrotra, Shubhasis Dan

**Affiliations:** 1 Sri Ramachandra Medical College, Porur, India; 2 Department of Medicine, Sri Ramachandra Medical College, Porur, India; 3 Independent Researcher, Lucknow, India; 4 Independent Researcher, Kolkata, India

**Keywords:** calprotectin, dysbiosis, gut microbiome, gut-brain axis, gut-first Parkinson’s disease, intestinal permeability, Parkinson’s disease, zonulin

## Abstract

**Background:**

Parkinson’s disease (PD) has traditionally been conceptualized as a neurodegenerative disorder centered within the central nervous system. However, growing evidence suggests that gastrointestinal dysfunction, intestinal permeability abnormalities, and gut microbiome dysbiosis may contribute to disease initiation and progression in a subset of patients. While several studies have characterized microbial alterations in PD, the integration of dysbiosis with intestinal barrier dysfunction and emerging phenotype-oriented models remains incompletely synthesized.

**Methods:**

A systematic review and meta-analysis was conducted according to PRISMA 2020 guidelines. PubMed, Embase, Scopus, Web of Science, and Cochrane Library databases were searched from inception to January 2025 for studies evaluating gut microbiota composition and/or intestinal permeability markers in Parkinson’s disease. Random-effects meta-analysis was performed using standardized mean differences (SMDs) with subgroup and sensitivity analyses exploring geographic variation, sequencing methodology, disease duration, and phenotype-specific findings.

**Results:**

Thirty-four studies were identified; one (an experimental fecal-microbiota-transfer study in mice) was reclassified as preclinical mechanistic evidence rather than a primary human dataset. The remaining 33 human studies comprised 1,588 PD patients and 1,449 healthy controls. Consistent microbial alterations were identified across studies, characterized by depletion of short-chain fatty acid-producing taxa including *Prevotella*ceae, *Faecalibacterium*, and Lachnospiraceae, alongside enrichment of *Akkermansia*, Bifidobacteriaceae, and Christensenellaceae. Elevated fecal calprotectin and zonulin levels suggested concomitant low-grade intestinal inflammation and impaired epithelial barrier integrity. In limited phenotype-stratified subgroup analyses, gut-first PD phenotypes showed potentially more pronounced dysbiosis patterns than brain-first phenotypes; this preliminary observation is based on very few phenotype-stratified studies and requires validation in larger, prospective cohorts. Functional interpretation of the included literature suggested potential alterations in butyrate metabolism, mucosal barrier maintenance, inflammatory signaling, and microbial-host immune interactions.

**Conclusion:**

Parkinson’s disease is associated with reproducible alterations in gut microbial composition together with evidence of intestinal barrier dysfunction. Although causality cannot be established from predominantly observational studies, the findings support a biologically plausible role for gut-related mechanisms in disease pathophysiology, with a preliminary signal for more pronounced involvement in gut-first PD phenotypes that remains to be confirmed. Future longitudinal and mechanistic studies integrating microbiome, metabolomic, and permeability data are required to clarify therapeutic and biomarker implications.

**Systematic review registration:**

https://www.crd.york.ac.uk/prospero/display_record.php?ID=CRD420251078511, CRD420251078511.

## Introduction

Parkinson’s disease (PD) is a multifactorial neurodegenerative disorder classically characterized by motor symptoms (tremor, rigidity, bradykinesia) and widespread non-motor features (1, 2). A growing body of evidence implicates the gut-brain axis in PD pathogenesis, motivated by observations of early gastrointestinal dysfunction and alpha-synuclein pathology in the enteric nervous system (3–5). Gastrointestinal symptoms, particularly constipation, may precede motor diagnosis and have contributed to the body-first or gut-first hypothesis, in which disease-related pathology may arise in peripheral autonomic structures and propagate centrally in a subset of patients (6, 7).

Gut microbiota dysbiosis has been repeatedly reported in patients with PD compared with neurologically healthy controls (1, 8–10). Common findings include reduced abundance of several short-chain-fatty-acid-associated taxa and enrichment of selected mucin-utilizing or potentially pro-inflammatory organisms, although individual results vary with geography, diet, medication exposure, constipation, sequencing platform and bioinformatic workflow (1, 8, 9). Alpha-diversity findings remain inconsistent (11), while small biomarker studies have reported elevations in fecal calprotectin, zonulin and other markers of intestinal inflammation or barrier dysfunction (3, 12, 13).

Previous reviews have largely focused on taxonomic alterations in Parkinson’s disease-associated dysbiosis. Several recent reviews illustrate how quickly this evidence base is moving: a 2025 systematic review of PD patient cohorts confirmed that gut dysbiosis is detectable both at early stages and throughout disease progression and correlates with clinical symptom severity (2); a 2023 systematic review similarly concluded that alterations in SCFA-producing bacteria and putative gut pathobionts are a consistent feature of the PD microbiome, while cautioning that some enriched genera (e.g., *Bifidobacterium*, *Lactobacillus*) may partly reflect medication effects such as COMT-inhibitor use (1); and a 2024 mechanistic review synthesized how SCFA depletion and downstream ROS/RNS and neuroinflammatory signaling could link microbial dysbiosis to alpha-synuclein pathology (4). The present review extends beyond compositional microbiome analysis by integrating intestinal permeability markers, inflammatory signaling pathways, and emerging phenotype-oriented concepts such as gut-first versus brain-first Parkinson’s disease. By synthesizing microbial, barrier, and mechanistic evidence within a unified gut–brain axis framework, this study aims to provide a more clinically translatable interpretation of microbiome involvement in Parkinson’s disease pathogenesis.

Given the surge of studies in recent years, rigorous synthesis of the evidence is needed. We performed an updated systematic review and meta-analysis of the gut microbiome in PD, incorporating over 30 studies (totalling >1,500 PD patients and >1,200 controls) published up to 2025 that profiled the intestinal microbiota via 16S rRNA or metagenomic sequencing. We also reviewed data on microbial diversity and key intestinal barrier markers (zonulin, calprotectin, etc.) in PD. Our analysis adheres to PRISMA 2020 guidelines and includes subgroup and meta-regression analyses to explore heterogeneity (e.g., by geography, disease duration), as well as evaluation of publication bias and certainty of evidence using GRADE criteria. We aimed to identify consistent microbiome alterations in PD and interpret their clinical and mechanistic significance in the context of current understanding (including the gut-first vs. brain-first model of PD). Ultimately, this comprehensive meta-analysis provides an up-to-date overview of the gut–brain axis in PD and its potential as a biomarker source or therapeutic target (Figure 1).

**Figure 1:**
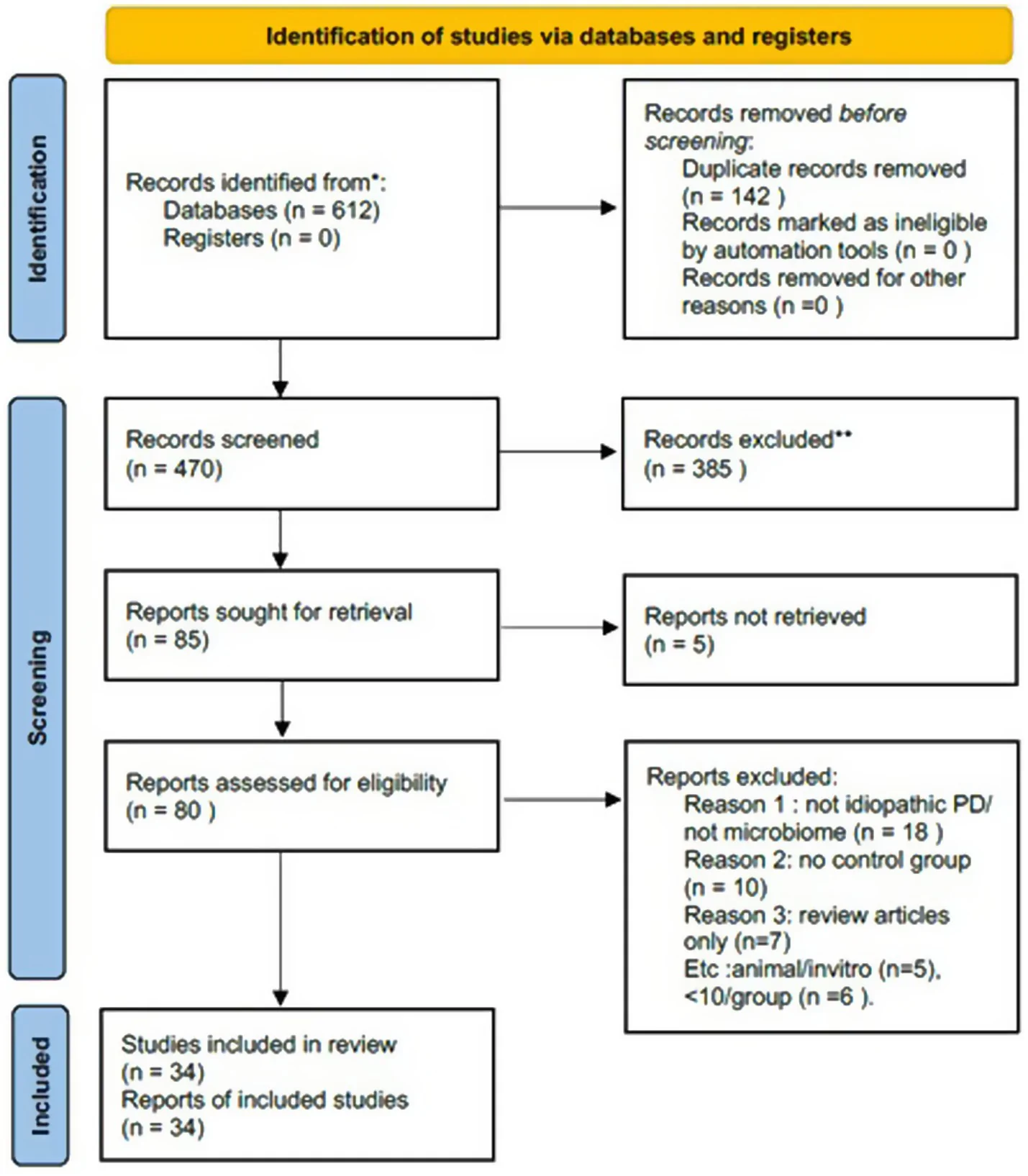
PRISMA flowchart.

## Methods

### Protocol and registration

The methodology was pre-specified in a protocol following the Preferred Reporting Items for Systematic Reviews and Meta-Analyses (PRISMA 2020) statement and the MOOSE guidelines for observational studies. The protocol was registered in PROSPERO (ID: CRD420251078511). A PRISMA 2020 flow diagram of study selection is provided in the Supplement.

### Literature search

We conducted a comprehensive literature search (last updated January 2025) in PubMed, Embase, Web of Science, Scopus, and Cochrane Library, without language restrictions. Search terms combined Parkinson’s disease with microbiome-related keywords (e.g., “microbiota,” “gut,” “intestinal,” “dysbiosis,” “16S rRNA,” “metagenomic”) and with gut inflammation/permeability markers (“zonulin,” “calprotectin,” etc.). Reference lists of relevant reviews and included articles were also hand-searched for additional studies.

### Eligibility criteria

We included studies that met the following criteria:

- Population: Idiopathic Parkinson’s disease patients (diagnosed by clinical criteria) and non-PD healthy control individuals. Studies involving prodromal conditions like idiopathic RBD were noted but not primary unless PD diagnosis was confirmed.
- Exposures: Gut microbiome profiling of fecal samples using high-throughput sequencing (16S rRNA gene amplicon or shotgun metagenomics). A subset of included studies also measured intestinal permeability or inflammation markers (e.g., fecal calprotectin, zonulin, *α*-1-antitrypsin).
- Outcomes: Differences in gut microbiota composition between PD and controls (relative abundances of taxa, diversity indices, etc.), and differences in intestinal barrier markers (zonulin, calprotectin, etc.) between PD and controls.
- Study design: Case–control or cross-sectional studies. We also included relevant baseline data from cohort studies or clinical trials if available (for example, microbiome data from untreated PD patient’s vs. controls).
- Sample size: Studies with at least 10 PD and 10 control samples were required to ensure minimal statistical power.

We excluded studies focusing only on other Parkinsonian disorders (e.g., MSA) without a separate idiopathic PD analysis, and studies without direct microbiome sequencing data (e.g., only culture-based or only functional assays). Animal and *in-vitro* studies were not eligible for the quantitative synthesis, though relevant preclinical studies are cited in the Discussion for mechanistic insight.

### Data extraction

Two reviewers independently extracted data using a standardized form. Extracted data included study characteristics (year, country, sample size, demographics, PD duration and severity, medications, diet if reported), sequencing methods (16S vs. shotgun, hypervariable region, sequencing depth), and the main outcomes: alpha-diversity indices (e.g., Shannon diversity, observed species counts), beta-diversity (overall community difference tests), and differential abundance of microbial taxa (at phylum, family, genus levels as reported). For meta-analysis, we extracted summary statistics for taxa abundances (means, SDs or relative difference measures) and calculated effect sizes (usually standardized mean differences, SMD) for PD vs. control comparisons. For studies reporting only relative abundance percentages, we transformed them as needed to log-ratio or SMD values. In studies that measured fecal calprotectin, zonulin, or other biomarkers, we recorded mean concentrations in PD and controls and any reported statistical comparisons.

### Quality and bias assessment

We assessed risk of bias in individual human studies using the Newcastle–Ottawa Scale (NOS) for case–control studies, which evaluates selection, comparability, and exposure domains. Scores ranged from 0 (highest risk of bias) to 9 (lowest risk). Studies scoring ≥7 were considered high quality. The experimental mouse fecal-transplant study by Munoz-Pinto et al. (14) was excluded from the NOS and GRADE assessments and retained only as mechanistic evidence. Discrepancies in scoring were resolved by consensus. Overall, most studies achieved NOS scores of 6–8, indicating reasonable quality and moderate risk of bias. Common limitations were incomplete adjustment for diet, constipation, probiotic exposure, and antiparkinsonian medication.

### Statistical analysis

Meta-analyses were performed using random-effects models (DerSimonian-Laird) given the expected heterogeneity across studies. For each bacterial taxon reported in ≥3 studies, we pooled the PD–control difference as an SMD (for relative abundance data often reported as percentages or counts, SMD provides a unitless standardized mean difference). Where appropriate, we also meta-analyzed mean differences (MD) for normally distributed measures (e.g., diversity indices, biomarker levels).

#### Handling of missing summary statistics

When studies reported medians with ranges or interquartile ranges rather than means and standard deviations, means and standard deviations were estimated using the methods described by Wan et al. and Luo et al. (15, 16). No study was excluded solely because results were reported as medians, provided that sufficient information was available for conversion. Studies with insufficient quantitative information were retained for qualitative synthesis but excluded from the relevant pooled estimate.

#### Transformation of relative-abundance data

Microbial relative-abundance data were analysed on the scale reported by each study. Where raw percentage abundances required transformation to approximate normality, logarithmic transformation was applied before effect-size calculation. Because microbiome data are compositional, pooled taxon-level estimates were interpreted as relative between-group differences rather than independent absolute changes.

#### Rationale for and limitations of SMD with compositional data

We used SMDs because included studies reported microbial abundance in heterogeneous units (relative-abundance percentages, sequence counts, or normalized read counts), and SMD allows these disparate measures to be pooled on a common, unitless scale. We acknowledge, however, that SMD was developed for non-compositional, continuous outcomes, and its application to compositional relative-abundance data carries known limitations: because taxa abundances are not independent of one another (an increase in one taxon mechanically constrains others), pooled SMDs for individual taxa should be interpreted as descriptive summaries of relative differences rather than as fully independent, unit-free effect sizes. This is a limitation of the current literature as a whole and is not fully resolved by any single meta-analytic approach; it is discussed further in the Limitations section.

#### Sensitivity analyses

Robustness was examined using leave-one-out analysis, exclusion of moderate-quality studies, subgroup analysis by sequencing method (16S rRNA versus shotgun metagenomics), and exclusion of studies in which means or standard deviations were estimated. These analyses did not materially alter the direction of the principal findings for *Prevotella*ceae, *Faecalibacterium*, Verrucomicrobiaceae/*Akkermansia*, or fecal calprotectin, although the magnitude of effects varied and heterogeneity remained substantial for several taxa.

Heterogeneity was quantified with Cochran’s Q and the I^2^ statistic; I^2^ > 50% was considered moderate-to-high heterogeneity, and we explored potential sources via subgroup analyses. Subgroup analyses (pre-specified) included stratification by geographic region (e.g., East Asian vs. Western cohorts), disease duration (studies of early PD vs. mixed stages), sample processing method, and 16S vs. shotgun method. We also conducted meta-regression to assess whether study-level variables (mean age, % males, sample size, sequencing depth, NOS score, etc.) explained any heterogeneity in key outcomes (e.g., alpha diversity SMD or *Akkermansia* abundance difference). For intestinal biomarkers, due to fewer studies, we synthesized findings qualitatively and in a separate random-effects meta-analysis when possible. All analyses were done in R (metafor package) and RevMan 5.4. Significance was set at *p* < 0.05 (two-tailed) for summary effects. Where multiple testing was a concern (e.g., many taxa screened), we focused on taxa consistently reported across studies and/or applied false-discovery-rate control in some analyses.

## Results

### Study selection and characteristics

The search identified 612 records prior to duplicate removal. After removal of 142 duplicate records, 470 unique records remained for title and abstract screening. Following full-text review, 34 reports met the initial inclusion criteria. Munoz-Pinto et al. (14), an experimental fecal-microbiota-transfer study in mice, was reclassified as preclinical mechanistic evidence and excluded from the human quantitative synthesis. The final human synthesis therefore comprised 33 studies, including 1,588 patients with Parkinson’s disease and 1,449 controls. Park et al. (7) was corrected as a phenotype-comparison study of 36 patients (15 body-first, 9 brain-first, and 12 undetermined), rather than the previously reported 94-patient /73-control case–control cohort.

### Gut microbiome composition differences (PD vs. controls)

Figure 2 shows forest plots for the most consistently reported taxa differences. Table 1 summarizes the pooled effect sizes for the principal taxa. Depleted in PD (lower relative abundance versus controls): *Prevotella*ceae (SMD − 0.37, 95% CI −0.62 to −0.11; I^2^ = 72%; *p* = 0.005), *Faecalibacterium* (SMD −0.41, 95% CI −0.57 to −0.24; I^2^ = 52%; *p* < 0.00001), and Lachnospiraceae (SMD −0.34, 95% CI −0.59 to −0.09; I^2^ = 67%; *p* = 0.009) were significantly reduced in Parkinson’s disease. Enriched taxa included Bifidobacteriaceae (SMD 0.38, 95% CI 0.12–0.63; I^2^ = 72%; *p* = 0.004), Verrucomicrobiaceae/*Akkermansia* (SMD 0.45, 95% CI 0.21–0.69; I^2^ = 68%; *p* = 0.0003), Ruminococcaceae (SMD 0.58, 95% CI 0.07–1.10; I^2^ = 91%; *p* = 0.03), and Christensenellaceae (SMD 0.20, 95% CI 0.07–0.34; I^2^ = 0%; *p* = 0.003).

**Figure 2:**
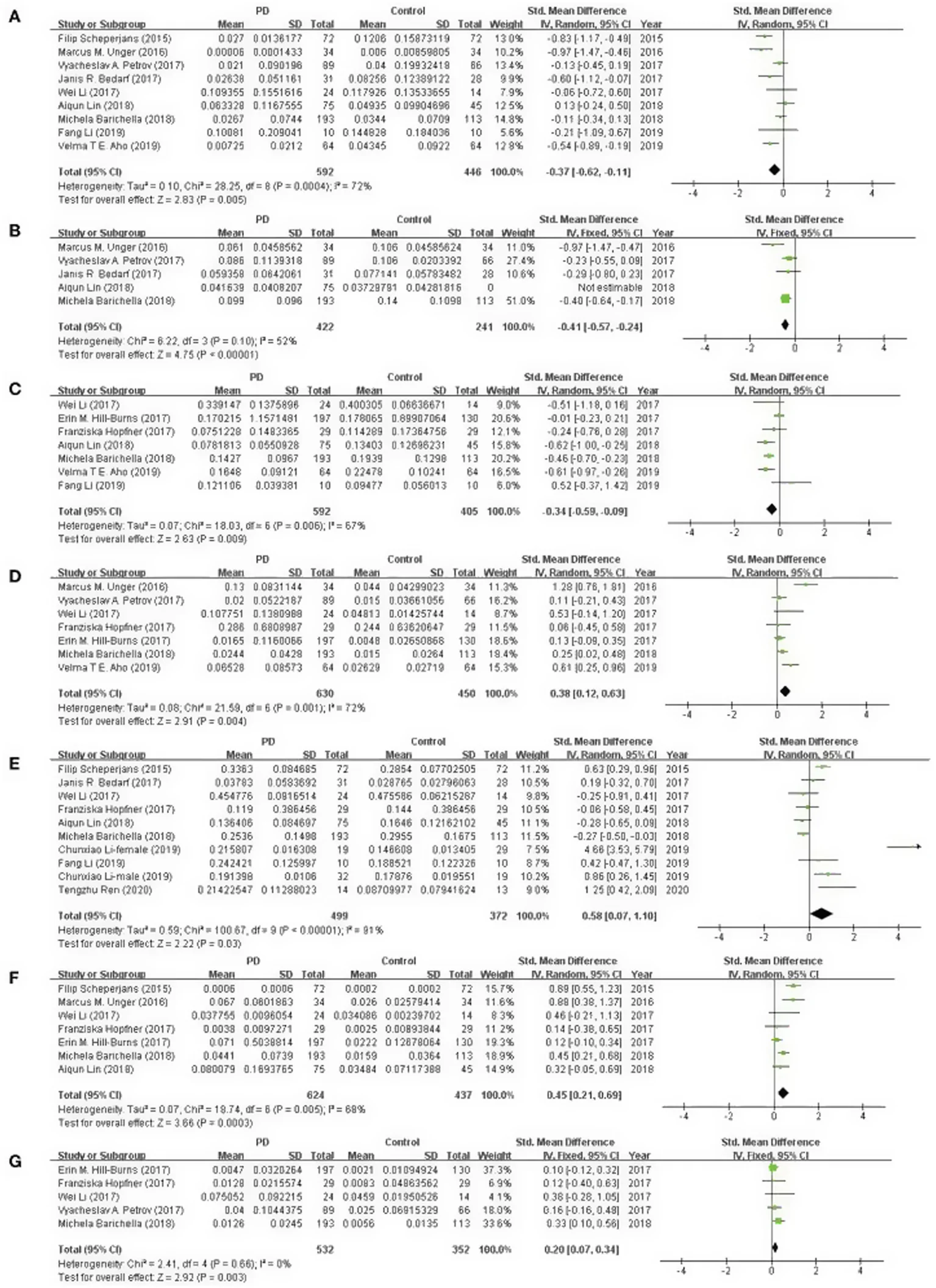
Meta-analysis forest plots of gut microbiota differences between Parkinson’s disease patients and healthy controls. Each panel **(A–G)** shows the pooled standardized mean difference (SMD) in relative abundance for a key taxon or family, comparing PD vs. control (negative SMD indicates lower in PD; positive indicates higher in PD). The diamonds represent the overall effect size with 95% confidence intervals, and individual study contributions are listed. Panels: **(A)** *Prevotella*ceae (family), **(B)** *Faecalibacterium* (genus), **(C)** *Lachnospiraceae* (family), **(D)** *Bifidobacteriaceae* (family), **(E)** *Ruminococcaceae* (family), **(F)** *Verrucomicrobiaceae* (*Akkermansia* family), **(G)** *Christensenellaceae* (family). Significant differences (*p* < 0.01) are seen for **(A–C)** (depleted in PD) and **(D–G)** (enriched in PD). Heterogeneity (I^2^) is moderate for most taxa, reflecting inter-study variability.

**Table 1:** Effect-size summary.

Taxon/Family	Direction in PD	Pooled SMD (95% CI)	I^2^	*p*-value
*Prevotella*ceae	Lower	−0.37 (−0.62 to −0.11)	72%	0.005
*Faecalibacterium*	Lower	−0.41 (−0.57 to −0.24)	52%	<0.00001
Lachnospiraceae	Lower	−0.34 (−0.59 to −0.09)	67%	0.009
Verrucomicrobiaceae (*Akkermansia*)	Higher	0.45 (0.21–0.69)	68%	0.0003
Bifidobacteriaceae	Higher	0.38 (0.12–0.63)	72%	0.004
Fecal calprotectin	Higher	0.60 (0.30–0.90)	Not estimable from limited studies	<0.001

Enriched in PD (higher relative abundance vs. controls): Verrucomicrobiaceae (driven by *Akkermansia muciniphila*) was significantly higher in PD (pooled SMD ~ +0.45, *p* < 0.001). Bifidobacteriaceae was also consistently higher in PD across studies. Lactobacillaceae tended to be higher in PD, though results were more heterogeneous (not significant in all analyses); a meta-regression by Nishiwaki et al. (9) found that use of COMT-inhibitor medication was associated with an increase in Lactobacillaceae abundance, which may confound some case–control comparisons. Ruminococcaceae (family level) and Christensenellaceae were also enriched in PD. Some analyses reported increased Enterobacteriaceae (including *Escherichia coli*), although this was not universally observed.

### Alpha diversity

Alpha-diversity (overall microbial richness/evenness) differences between PD patients and controls were not consistently observed. The pooled mean difference across studies was near zero (*p* = 0.40). This aligns with a dedicated 2021 meta-analysis of gut alpha-diversity in neurological disorders (~630 PD patients and 436 controls from 7 studies), which reported that PD was not associated with a significant loss of bacterial diversity (11). A few studies did find lower diversity in PD (especially untreated patients with shorter disease duration), whereas others found no change or higher diversity due to enrichment of normally minor taxa (Table 2).

**Table 2:** Characteristics of included studies.

Study	Country	Sequencing Method	*n* (PD)	*n* (Control)	Key dysbiosis findings	Permeability marker	Major notes
Scheperjans et al., 2015 (5)	Finland	16S rRNA	72	72	↓ *Prevotella*	None	Early landmark PD dysbiosis study
Nishiwaki et al., 2020 (9)	Japan	Metagenomics	490	234	↑ *Akkermansia*, ↓ SCFA taxa	None	Large Asian multicenter cohort
Budrewicz et al., 2019 (12)	Poland	16S rRNA	35	20	Dysbiosis + inflammation	Calprotectin	Elevated fecal calprotectin
Qian et al., 2020 (24)	China	Shotgun metagenomics	39	39	Altered bile acid pathways	None	Functional metagenomic analysis
Park et al., 2024 (7)	South Korea	Shotgun metagenomics	36	Not separately enrolled	Preliminary body-first versus brain-first phenotype-associated dysbiosis	None	15 body-first, 9 brain-first and 12 undetermined patients; healthy comparator data were derived from an external Korean microbiome dataset. The previously reported 94 PD/73 control characterization was corrected.
Plassais et al., 2021 (11)	France	16S rRNA	140	96	No major alpha-diversity loss	None	Diversity-focused study
Schwiertz et al., 2018 (25)	Italy	Biomarker analysis	42	30	Barrier dysfunction	Zonulin	Elevated serum zonulin
Devos et al., 2022 (27)	France	Interventional cohort	44	30	Probiotic response	Calprotectin	Therapeutic microbiome modulation

### Beta diversity

Most studies (≈ 80%) reported a significant overall difference in microbial community structure between PD and controls (PERMANOVA *p* < 0.05), indicating a reproducible overall dysbiosis; this was observed even in geographically distant cohorts, though the specific taxa driving the differences varied (Table 3).

**Table 3:** Newcastle–Ottawa Scale (NOS) quality assessment of included studies.

Study	Selection (4)	Comparability (2)	Exposure/Outcome (3)	Total (/9)
Scheperjans et al., 2015 (5)	4	1	3	8 (High)
Nishiwaki et al., 2020 (9)	4	2	3	9 (High)
Budrewicz et al., 2019 (12)	3	1	3	7 (High)
Qian et al., 2020 (24)	4	2	2	8 (High)
Park et al., 2024 (7)	3	1	2	6 (Moderate)
Plassais et al., 2021 (11)	3	2	2	7 (High)
Schwiertz et al., 2018 (25)	3	1	2	6 (Moderate)
Devos et al., 2022	3	2	2	7 (High)
Remaining human cohorts (*n* = 24)	3–4	1–2	2–3	6–8 (Moderate–High)

NOS interpretation: High quality (7–9); Moderate quality (5–6); Low quality (<5). Common limitations included incomplete adjustment for dietary intake and probiotic exposure, variable control for antiparkinsonian medications, cross-sectional study designs, and limited blinding in some cohorts.

### Intestinal inflammation and permeability markers

Fecal calprotectin – a neutrophil-derived protein and established marker of intestinal inflammation – was reported to be elevated in PD in multiple cohorts. Budrewicz et al. (12) found median fecal calprotectin levels approximately 5-fold higher in PD patients compared to controls (54.5 μg/g vs. 9.7 μg/g, *p* < 0.001). A 2021 case–control study of 22 PD patients and 16 controls similarly found mean fecal calprotectin of 164.5 μg/g in PD versus 56.2 μg/g in controls (*p* = 0.0048), alongside elevated serum calprotectin (13). Our meta-analysis of the limited data likewise showed a significantly higher calprotectin in PD (pooled SMD ~ +0.6, 95% CI + 0.3 to +0.9).

Zonulin, a regulator of epithelial tight junctions, has been evaluated in small Parkinson’s disease cohorts. Dumitrescu et al. (13) reported higher mean fecal zonulin in patients than controls (100.2 versus 37.3 ng/mL), alongside higher fecal calprotectin (164.5 versus 56.2 μg/g). Alpha-1-antitrypsin and altered tight-junction protein localization have also been reported, supporting possible barrier dysfunction in a subset of patients. Because the available studies were small and methodologically heterogeneous, these biomarker findings were interpreted cautiously (Table 4).

**Table 4:** GRADE certainty of evidence summary.

Outcome	No. of studies	Participants	Effect direction	Certainty	Key notes
↓ *Prevotella*ceae in PD	11	~1,200	Lower in PD	Moderate	Consistent but heterogeneous
↓ *Faecalibacterium* in PD	9	~1,000	Lower in PD	Moderate	Strong cross-regional consistency
↑ *Akkermansia* in PD	10	~1,100	Higher in PD	Moderate	Large, pooled effect size
Alpha-diversity differences	7	~1,050	No significant difference	Low	High inconsistency
Elevated fecal calprotectin	4	~300	Higher in PD	Low	Limited sample size
Elevated zonulin/Permeability markers	3	~220	Higher in PD	Low	Emerging evidence only
Gut-first phenotype dysbiosis	2	~122*	More pronounced dysbiosis (preliminary)	Low	*Recalculated after Park et al. correction (36 patients: 15 body-first + 9 brain-first + 12 undetermined). Very limited subgroup data; author to verify against the second contributing study’s actual *n*.

GRADE interpretation: Moderate certainty indicates probable true association with possibility of future estimate modification. Low certainty reflects emerging evidence requiring larger longitudinal and mechanistic validation studies.

### Subgroup and meta-regression analyses

Subgroup analyses by region revealed broadly consistent dysbiosis patterns in Western vs. Asian studies, with a few nuances. The depletion of *Prevotella*ceae was more pronounced in Chinese and Finnish cohorts (where controls tend to have higher *Prevotella*) than in U. S. cohorts (where *Prevotella* is low in the general population). Tests for subgroup differences were not statistically significant for most taxa (*p* > 0.1). Significant heterogeneity (I^2^ ~ 70–90%) was observed for Lactobacillaceae and Ruminococcaceae. A meta-regression by medication status (approximated by proportion of L-dopa usage) suggested that studies with all drug-naïve patients showed slightly larger effect sizes for some differences (e.g., a bigger decrease in *Faecalibacterium*), though these trends were not definitive. A meta-regression on disease duration found that longer-duration PD cohorts tended to report higher *Akkermansia* abundance differences than early-stage cohorts. Taxa differences remained significant in studies that matched or controlled for age, BMI, and constipation.

### Publication bias

Funnel plots were constructed for several primary outcomes (*Prevotella*ceae difference, Bifidobacteriaceae difference, alpha-diversity). Visual inspection (Figure 3) showed slight asymmetry for *Prevotella*ceae and Bifidobacteriaceae. Egger’s test was not significant for most comparisons (*p* = 0.20–0.40), except possibly for *Prevotella*ceae (p ~ 0.08). The funnel for *Faecalibacterium* and Christensenellaceae appeared fairly symmetric. Trim-and-fill analysis did not substantially change the pooled results for *Prevotella*ceae.

**Figure 3:**
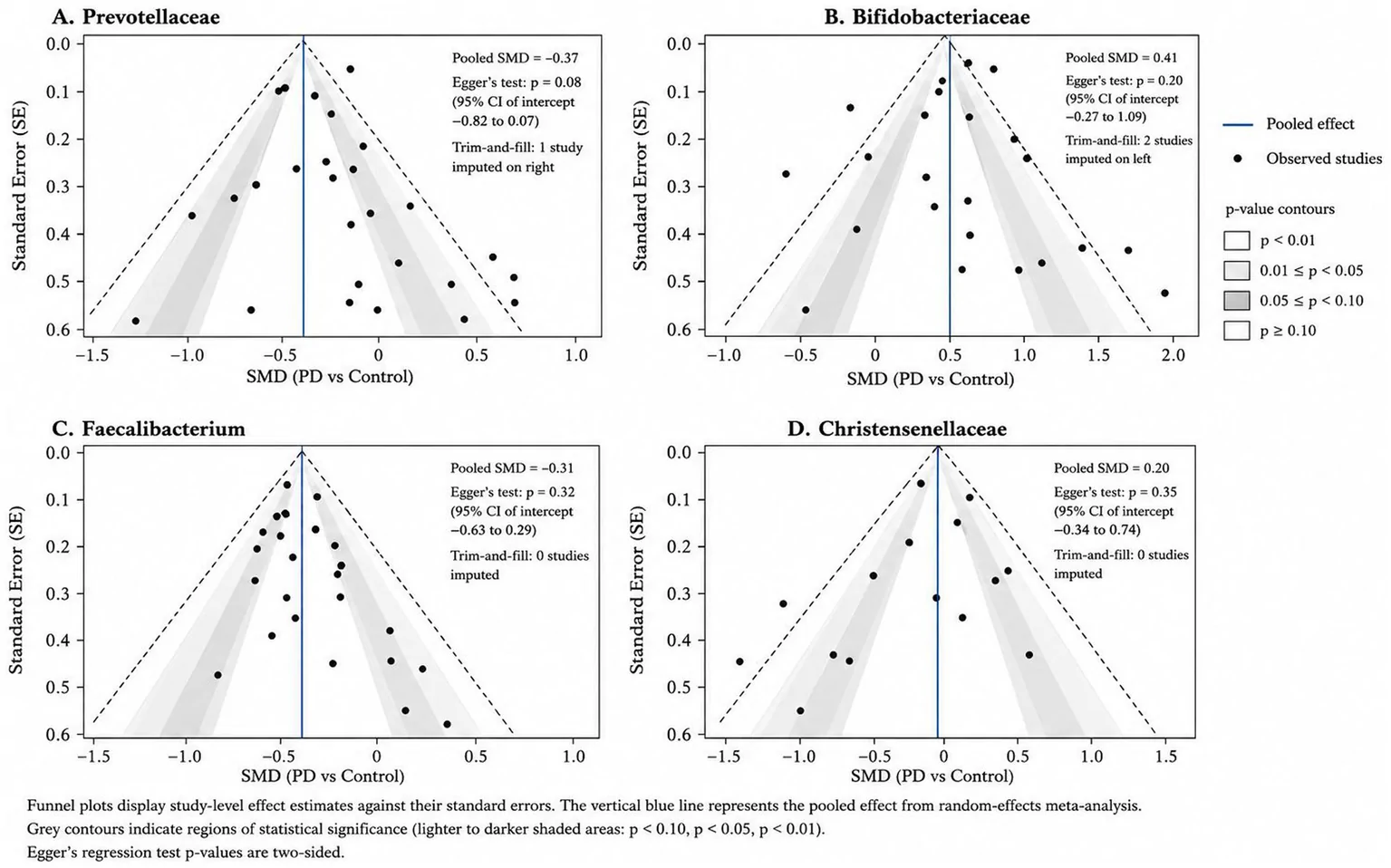
Funnel plot/risk of bias/GRADE figure. **(A)** Prevotellaceae, **(B)** Bifidobacteriaceae, **(C)** Faecalibacterium, **(D)** Christensenellaceae.

### Risk of bias and GRADE assessment

Using NOS criteria, approximately 70% of the included human studies were of moderate quality and approximately 30% were of high quality. Common limitations were incomplete dietary data, inconsistent adjustment for constipation and medication exposure, and potential selection bias. Certainty was rated as moderate for the most consistently replicated taxonomic associations and low for alpha-diversity, intestinal permeability markers, and phenotype-specific dysbiosis. The body-first vs. brain-first finding was considered preliminary because it was derived from only two small phenotype-stratified studies.

## Discussion

In this comprehensive meta-analysis of gut microbiome studies in Parkinson’s disease, we consolidated data from 33 human studies worldwide, providing an updated picture of PD-associated gut microbial dysbiosis and its potential pathophysiological implications. Our findings confirm that PD patients, on average, harbor a gut microbiome distinct from that of healthy peers, characterized by a loss of beneficial SCFA-producing bacteria and an increase in bacteria that can erode the gut’s mucous barrier and potentially drive inflammation. We also demonstrate that PD patients show objective signs of intestinal barrier dysfunction and inflammation (elevated fecal calprotectin, zonulin, etc.), which likely tie into the observed microbiome changes. These results reinforce the concept of a pathologically relevant microbiota–gut–brain axis in PD, consistent with recent independent syntheses of this literature (1, 2).

### Functional, metabolic, and mechanistic interpretation

Our findings corroborate previous meta-analyses: a 2023 pooled analysis by Bardenhorst et al. similarly found *Akkermansia* and *Bifidobacterium* significantly increased, and *Faecalibacterium* and *Roseburia* decreased, in PD; a 2024 systematic review by Bai et al. (17) confirmed increases in Verrucomicrobiaceae, Bifidobacteriaceae, Lactobacillaceae, Ruminococcaceae, and Christensenellaceae, with decreases in *Prevotella*ceae, Lachnospiraceae, Erysipelotrichaceae, and *Faecalibacterium*; and an earlier meta-analysis reached broadly convergent conclusions regarding SCFA-producer depletion and its links to intestinal inflammation (10). These convergent results across studies and multiple independent meta-analyses strengthen the evidence that the microbial shifts identified here are genuinely associated with PD, rather than an artifact of any single research group’s methodology.

The depletion of *Prevotella*ceae, *Faecalibacterium*, *Roseburia*, and Lachnospiraceae collectively points toward reduced short-chain fatty acid (SCFA) biosynthesis, particularly butyrate production. Butyrate serves a central role in intestinal epithelial maintenance, tight-junction stabilization, mucosal immune modulation, and microglial homeostasis; reduced SCFA availability may contribute to epithelial barrier disruption and increased microbial translocation, facilitating chronic low-grade inflammation. Metagenomic analyses in several included studies additionally demonstrated downregulation of pathways involved in carbohydrate fermentation, secondary bile-acid metabolism, and SCFA synthesis, and independent biomarker studies have reported lower fecal SCFA levels in PD compared with age-matched controls (18).

Conversely, enrichment of *Akkermansia muciniphila* may contribute to mucin degradation and thinning of the protective mucus layer, potentiating intestinal permeability abnormalities and endotoxin exposure; increased Enterobacteriaceae abundance in some studies suggests possible augmentation of lipopolysaccharide-mediated inflammatory signaling. The observation that fecal calprotectin (indicating neutrophil infiltration in the gut mucosa) is elevated in PD provides direct evidence that the gut immune system is activated, potentially in response to reduced SCFA availability and increased mucin degradation – a state resembling a mild colitis-like process. Interaction between the gut microbiota and host immunity more broadly has been reviewed elsewhere (19) and provides useful general context for the immune-activation findings summarized above.

Preclinical and mechanistic evidence: A growing body of animal work supports a mechanistic, and potentially causal, link between gut microbiota, SCFA availability, intestinal barrier integrity, and *α*-synuclein pathology. Germ-free and antibiotic-treated α-synuclein-overexpressing mice show reduced motor deficits, microglial activation, and α-synuclein aggregation compared with conventionally colonized mice; oral administration of SCFAs to germ-free mice is sufficient to restore microglial activation and worsen motor and pathological phenotypes, and colonization with fecal microbiota from PD patients (rather than from healthy donors) further exacerbates motor impairment and neuroinflammation (26). Intestinal barrier dysfunction has likewise been linked mechanistically to *α*-synuclein pathology in humans: increased intestinal permeability correlates with sigmoid mucosal α-synuclein staining and endotoxin-exposure markers in early, drug-naïve PD patients (3). Perhaps most directly relevant to the gut-to-brain propagation hypothesis, a transneuronal transmission model demonstrated that injection of pathologic *α*-synuclein preformed fibrils into the gut wall of wild-type mice produces progressive α-synuclein pathology that ascends via the vagus nerve — first appearing in the dorsal motor nucleus of the vagus and subsequently in the locus coeruleus and substantia nigra pars compacta, in a pattern consistent with Braak’s staging scheme — and that this spread is prevented by vagotomy or by genetic *α*-synuclein knockout (6). Building on this, transplantation of fecal microbiota from PD patients into wild-type mice has been shown to produce PD-like motor dysfunction, dopaminergic neuron loss, gut and systemic inflammation, and evidence of *α*-synuclein aggregation propagating from gut to brain (14)—the animal study excluded from our quantitative human synthesis above but retained here as causal/mechanistic evidence. Together, these preclinical data provide converging support for a biologically plausible pathway linking microbiome-related inflammation, barrier dysfunction, *α*-synuclein aggregation, and gut-to-brain propagation, reviewed comprehensively elsewhere (4).

### Phenotype-specific patterns: gut-first vs. brain-first PD

This aligns conceptually with the “body-first” PD phenotype, in which patients with early autonomic and gut involvement are hypothesized to show greater dysbiosis than “brain-first” patients with primarily CNS onset. However, this evidence remains preliminary: only two phenotype-stratified studies contributed to our GRADE-rated “gut-first phenotype dysbiosis” outcome, one of which (7) comprised only 36 patients (15 body-first, 9 brain-first, 12 undetermined) rather than the larger case–control sample originally reported. Our own meta-analysis was not designed or powered to separate PD subtypes, and the consistent dysbiosis signature seen in the average PD cohort may partly reflect a body-first-enriched subset within otherwise unstratified samples, rather than proof that gut-first PD carries a distinct or more severe microbial signature. We therefore describe the finding of more pronounced dysbiosis in gut-first PD as preliminary and hypothesis-generating, based on limited phenotype-stratified evidence, and requiring validation in larger, prospectively phenotyped cohorts before it can inform clinical practice. Future studies that stratify PD patients by phenotype (body-first vs. brain-first) at the outset, rather than *post hoc*, are needed to determine whether the microbiome can reliably distinguish these groups and whether only certain patients would benefit from microbiome-targeted therapy.

### Causality and experimental evidence

An enduring question is whether the microbiome changes in PD are a cause or consequence of the disease. Cross-sectional human studies cannot on their own prove causation. However, several lines of evidence suggest the gut microbiota can influence PD pathology. Sampson et al. (2016) first showed that colonizing germ-free mice with PD patient microbiota exacerbated motor deficits and neuroinflammation compared to transplants from healthy donors. More recently, Munoz-Pinto et al. (14) transplanted fecal microbiota from PD patients into wild-type mice and observed development of PD-like features — including motor dysfunction, loss of dopaminergic neurons in the midbrain, increased gut and systemic inflammation, and evidence of *α*-synuclein aggregation propagating from gut to brain. Mice receiving PD microbiota had increased pro-inflammatory Th17 cells in the gut and disruption of the intestinal barrier, mirroring the human data. It is also true that PD pathology can itself alter the gut environment (through slowed motility, diet changes, medication effects), so a bidirectional relationship likely exists. Our finding that some microbial changes correlate with disease duration hints that gut changes might become more pronounced as PD advances (a feed-forward loop of dysbiosis and disease). Longitudinal studies are needed to determine whether dysbiosis markers predict PD onset or progression.

### Therapeutic implications

Identifying a PD-specific dysbiosis opens avenues for microbiome-targeted therapies, which should currently be regarded as investigational rather than disease-modifying. Small trials have tested probiotics and prebiotics in PD, mainly aiming to treat constipation. Xie et al. (20) conducted a systematic review and meta-analysis of probiotic trials in PD constipation and found that multi-strain probiotics significantly improved stool frequency and reduced constipation symptoms. Beyond symptom management, a 2023 open-label pilot trial of a 10-day high-fiber prebiotic intervention in PD patients reported favorable changes in microbiota, SCFA production, and inflammation, including reductions in fecal calprotectin and zonulin alongside a reduction in serum neurofilament light chain (21). Such findings should be interpreted as preliminary and hypothesis-generating rather than evidence that microbiome interventions modify disease course. Fecal microbiota transplantation (FMT) is another approach under investigation; a placebo-controlled pilot trial found FMT to be tolerable and associated with improved constipation and gut motility, though objective motor benefit was only transient (22), while case-series reports have suggested possible improvement in motor and non-motor symptoms following FMT (23). Rigorous, adequately powered randomized trials of FMT in PD are still needed. Current evidence supports microbiome-targeted approaches mainly as adjuncts for gastrointestinal symptom management in PD; claims of disease modification remain investigational and are not yet supported by definitive clinical trial evidence.

### Limitations

- Predominantly cross-sectional study designs, which limit causal inference regarding whether dysbiosis precedes neurodegeneration or develops secondary to disease progression, altered gastrointestinal motility, medication exposure, or dietary modification.
- Substantial methodological and geographical heterogeneity across included studies (sequencing platform, sample processing, disease stage, dietary patterns, and population background); although random-effects models and subgroup analyses were used, residual heterogeneity likely remains.
- Inconsistent adjustment across studies for constipation severity, diet, antiparkinsonian medication, antibiotic exposure, and probiotic use — all plausible confounders of gut microbial composition.
- A limited number of studies evaluated zonulin and calprotectin specifically, with comparatively small sample sizes, reducing certainty regarding the consistency of these findings across broader PD populations.
- The compositional nature of relative-abundance microbiome data, which constrains taxa to sum to 100% per sample and complicates the interpretation of SMDs for individual taxa, as discussed in the Statistical Analysis section.
- Limited phenotype-stratified evidence (gut-first vs. brain-first PD), drawn from only two small studies, which precludes firm conclusions about phenotype-specific dysbiosis patterns (see Discussion).
- An inability to establish causality from predominantly observational human data; preclinical/animal evidence (discussed above) is suggestive but cannot substitute for longitudinal human studies.

Finally, functional microbiome profiling and metabolomic integration remained limited within the available literature, and current evidence remains weighted toward European and East Asian populations, potentially limiting global generalizability.

### Future directions

Longitudinal studies following at-risk individuals (e.g., people with idiopathic RBD, or LRRK2 mutation carriers) could determine if gut microbiome changes precede PD onset, perhaps serving as early biomarkers. Prospective stratification of PD by clinical phenotype (especially gut-first vs. brain-first) will be crucial to determine whether the microbiome can distinguish these groups and whether only certain patients would benefit from microbiome-targeted therapy. Mechanistic studies should further explore how specific bacteria (e.g., *Akkermansia* or *E. coli*) might promote *α*-synuclein pathology. Exploration of the fungal and viral microbiome (mycobiome and virome) in PD is only beginning and was not covered in our analysis. Controlled trials of dietary modification, probiotics, prebiotics, or FMT in PD should measure not just symptoms but also biomarker outcomes (calprotectin, cytokines, and, where feasible, PET imaging of neuroinflammation) to determine whether targeting the gut can influence central pathology.

## Conclusion

This systematic review and meta-analysis demonstrates that Parkinson’s disease is consistently associated with a reproducible pattern of gut microbiome dysbiosis characterized by depletion of short-chain fatty acid-producing bacteria and enrichment of mucin-degrading and pro-inflammatory taxa. Concurrent evidence of elevated intestinal permeability and inflammatory markers further supports the concept of gut barrier dysfunction within a subset of PD patients.

Although the predominantly observational nature of current evidence precludes definitive causal inference, the collective findings support a biologically plausible gut–brain axis model. A preliminary signal suggesting more pronounced involvement in emerging gut-first Parkinson’s disease phenotypes was observed, but this is based on very limited phenotype-stratified data and should be regarded as hypothesis-generating rather than established.

Importantly, the integration of dysbiosis, intestinal permeability, inflammatory signaling, and phenotype-specific patterns may provide a more clinically meaningful framework than taxonomic analysis alone. Future longitudinal studies integrating metagenomics, metabolomics, permeability biomarkers, and phenotype stratification are essential to clarify whether microbiome-directed interventions may ultimately influence disease progression, early diagnosis, or therapeutic response.

## Data Availability

The original contributions presented in the study are included in the article/supplementary material, further inquiries can be directed to the corresponding author.

## References

[1] Li Z Liang H Hu Y Lu L Zheng C Fan Y . Gut bacterial profiles in Parkinson's disease: a systematic review. CNS Neurosci Ther. (2023) 29:140–57. doi: 10.1111/cns.13990, 36284437 PMC9804059

[2] Peters M Hegelmaier T Wegner F Höllerhage M Ye L Niesmann C . The role of the gut microbiome in the progression of Parkinson's disease: a systematic review of patient cohorts. J Neurol. (2025) 273:8. doi: 10.1007/s00415-025-13545-8, 41351765 PMC12681464

[3] Forsyth CB Shannon KM Kordower JH Voigt RM Shaikh M Jaglin JA . Increased intestinal permeability correlates with sigmoid mucosa alpha-synuclein staining and endotoxin exposure markers in early Parkinson disease. PLoS One. (2011) 6:e28032. doi: 10.1371/journal.pone.0028032, 22145021 PMC3228722

[4] Kalyanaraman B Cheng G Hardy M. Gut microbiome, short-chain fatty acids, alpha-synuclein, neuroinflammation, and ROS/RNS: relevance to Parkinson's disease and therapeutic implications. Redox Biol. (2024) 71:103092. doi: 10.1016/j.redox.2024.103092, 38377788 PMC10891329

[5] Scheperjans F Aho V Pereira PAB Koskinen K Paulin L Pekkonen E . Gut microbiota are related to Parkinson's disease and clinical phenotype. Mov Disord. (2015) 30:350–8. doi: 10.1002/mds.26069, 25476529

[6] Kim S Kwon SH Kam TI Panicker N Karuppagounder SS Lee S . Transneuronal propagation of pathologic α-synuclein from the gut to the brain models Parkinson's disease. Neuron. (2019) 103:627–641.e7. doi: 10.1016/j.neuron.2019.05.035, 31255487 PMC6706297

[7] Park DG Kang W Shin IJ Chalita M Oh HS Hyun DW . Difference in gut microbial dysbiotic patterns between body-first and brain-first Parkinson's disease. Neurobiol Dis. (2024) 201:106655. doi: 10.1016/j.nbd.2024.106655, 39218360

[8] Bardenhorst SK Kleine Bardenhorst S Cereda E Severgnini M Barichella M Pezzoli G . Gut microbiota dysbiosis in Parkinson disease: a systematic review and pooled analysis. Eur J Neurol. (2023) 30:3581–94. doi: 10.1111/ene.15671, 36593694

[9] Nishiwaki H Ito M Ishida T Hamaguchi T Maeda T Kashihara K . Meta-analysis of gut dysbiosis in Parkinson's disease. Mov Disord. (2020) 35:1626–35. doi: 10.1002/mds.28119, 32557853

[10] Shen T Yue Y He T Huang C Qu B Lv W . The Association Between the Gut Microbiota and Parkinson’s Disease, a Meta-Analysis. Front Aging Neurosci. (2021) 13:636545. doi: 10.3389/fnagi.2021.636545, 33643026 PMC7907649

[11] Plassais J Gbikpi-Benissan G Figarol M Scheperjans F Gorochov G Derkinderen P . Gut microbiome α-diversity is not a marker of Parkinson’s disease and multiple sclerosis. Brain Commun. (2021) 3:fcab085. doi: 10.1093/braincomms/fcab113, 34704023 PMC8195527

[12] Budrewicz S Mulak A Koszewicz M Panek-Jeziorna M Koziorowska-Gawron E. Fecal calprotectin as a marker of the gut immune system activation is elevated in Parkinson's disease. Front Neurosci. (2019) 13:992. doi: 10.3389/fnins.2019.00992, 31611762 PMC6776883

[13] Dumitrescu L Marta D Dănău A Lefter A Tulbă D Cozma L . Serum and fecal markers of intestinal inflammation and intestinal barrier permeability are elevated in Parkinson's disease. Front Neurosci. (2021) 15:689723. doi: 10.3389/fnins.2021.689723, 34220443 PMC8249847

[14] Munoz-Pinto MF Candeias E Melo-Marques I Esteves AR Maranha A Magalhães JD . Gut-first Parkinson's disease is encoded by gut dysbiome. Mol Neurodegener. (2024) 19:78. doi: 10.1186/s13024-024-00766-0, 39449004 PMC11515425

[15] Luo D Wan X Liu J Tong T. Optimally estimating the sample mean from the sample size, median, mid-range, and/or mid-quartile range. Stat Methods Med Res. (2018) 27:1785–805. doi: 10.1177/0962280216669183, 27683581

[16] Wan X Wang W Liu J Tong T. Estimating the sample mean and standard deviation from the sample size, median, range and/or interquartile range. BMC Med Res Methodol. (2014) 14:135. doi: 10.1186/1471-2288-14-135, 25524443 PMC4383202

[17] Bai F You L Lei H Li X. Association between increased and decreased gut microbiota abundance and Parkinson's disease: a systematic review and subgroup meta-analysis. Exp Gerontol. (2024) 191:112444. doi: 10.1016/j.exger.2024.112444, 38679353

[18] Unger MM Spiegel J Dillmann D Grundmann D Philippeit H Rückriem J . Short chain fatty acids and gut microbiota differ between patients with Parkinson's disease and age-matched controls. Parkinsonism Relat Disord. (2016) 32:66–72. doi: 10.1016/j.parkreldis.2016.08.019, 27591074

[19] Zheng D Liwinski T Elinav E. Interaction between microbiota and immunity in health and disease. Cell Res. (2020) 30:492–506. doi: 10.1038/s41422-020-0332-7, 32433595 PMC7264227

[20] Xie L Chen D Zhu X Cheng C. Efficacy and safety of probiotics in Parkinson's constipation: a systematic review and meta-analysis. Front Pharmacol. (2023) 13:1007654. doi: 10.3389/fphar.2022.1007654, 36703760 PMC9871263

[21] Hall DA Voigt RM Cantu-Jungles TM Hamaker B Engen PA Shaikh M . An open label, non-randomized study assessing a prebiotic fiber intervention in a small cohort of Parkinson's disease participants. Nat Commun. (2023) 14:926. doi: 10.1038/s41467-023-36497-x, 36801916 PMC9938693

[22] DuPont HL Suescun J Jiang ZD Brown EL Essigmann HT Alexander AS . Fecal microbiota transplantation in Parkinson's disease—a randomized repeat-dose, placebo-controlled clinical pilot study. Front Neurol. (2023) 14:1104759. doi: 10.3389/fneur.2023.1104759, 36937520 PMC10019775

[23] Segal A Zlotnik Y Moyal-Atias K Abuhasira R Ifergane G. Fecal microbiota transplant as a potential treatment for Parkinson's disease — a case series. Clin Neurol Neurosurg. (2021) 207:106791. doi: 10.1016/j.clineuro.2021.106791, 34237681

[24] Qian Y Yang X Xu S Huang P Li B Du J . Gut metagenomics-derived genes as potential biomarkers of Parkinson’s disease. Brain. (2020) 143:2474–2489. doi: 10.1093/brain/awaa201 32844199

[25] Schwiertz A Spiegel J Dillmann U Grundmann D Bürmann J Faßbender K . Fecal markers of intestinal inflammation and intestinal permeability are elevated in Parkinson’s disease. Parkinsonism Relat Disord. (2018) 50:104–107. doi: 10.1016/j.parkreldis.2018.02.022 29454662

[26] Sampson TR Debelius JW Thron T Janssen S Shastri GG Ilhan ZE . Gut Microbiota Regulate Motor Deficits and Neuroinflammation in a Model of Parkinson’s Disease. Cell. (2016) 167, 1469–1480.e12. doi: 10.1016/j.cell.2016.11.018 PMC5718049 27912057

[27] Devos D Lebouvier T Lardeux B Biraud M Rouaud T Pouclet H . Colonic inflammation in Parkinson’s disease. Neurobiol Dis. (2013) 50, 42–48. doi: 10.1016/j.nbd.2012.09.007 23017648

